# Process evaluation of a community-based adolescent obesity prevention project in Tonga

**DOI:** 10.1186/1471-2458-11-284

**Published:** 2011-05-09

**Authors:** Kalesita F Fotu, Marj M Moodie, Helen M Mavoa, Siosifa Pomana, Jimaima T Schultz, Boyd A Swinburn

**Affiliations:** 1 School of Public Health, Fiji School of Medicine, Fiji; 2 Deakin Health Economics, Deakin University, Australia; 3 WHO Collaborating Centre for Obesity Prevention, Deakin University, Australia

## Abstract

**Background:**

The rising burden of obesity in Tonga is alarming. The promotion of healthy behaviours and environments requires immediate urgent action and a multi-sectoral approach. A three-year community based study titled the Ma'alahi Youth Project (MYP) conducted in Tonga from 2005-2008 aimed to increase the capacity of the whole community (schools, churches, parents and adolescents) to promote healthy eating and regular physical activity and to reduce the prevalence of overweight and obesity amongst youth and their families. This paper reflects on the process evaluation for MYP, against a set of Best Practice Principles for community-based obesity prevention.

**Methods:**

MYP was managed by the Fiji School of Medicine. A team of five staff in Tonga were committed to planning, implementation and evaluation of a strategic plan, the key planks of which were developed during a two day community workshop. Intervention activities were delivered in villages, churches and schools, on the main island of Tongatapu. Process evaluation data covering the resource utilisation associated with all intervention activities were collected, and analysed by dose, frequency and reach for specific strategies. The action plan included three standard objectives around capacity building, social marketing and evaluation; four nutrition; two physical activity objectives; and one around championing key people as role models.

**Results:**

While the interventions included a wide mix of activities straddling across all of these objectives and in both school and village settings, there was a major focus on the social marketing and physical activity objectives. The intervention reach, frequency and dose varied widely across all activities, and showed no consistent patterns.

**Conclusions:**

The adolescent obesity interventions implemented as part of the MYP program comprised a wide range of activities conducted in multiple settings, touched a broad spectrum of the population (wider than the target group), but the dose and frequency of activities were generally insufficient and not sustained. Also the project confirmed that, while the MYP resulted in increased community awareness of healthy behaviours, Tonga is still in its infancy in terms of conducting public health research and lacks research infrastructure and capacity.

## Background

The prevalence of obesity and associated diseases such as diabetes and hypertension is very high and increasing in the Pacific [1-3]. Tonga is among the top five countries worldwide in terms of obesity, [4] with 84% of Tongan males and 93% of females classified as overweight or obese [5]. During the last 30 years, Tonga has experienced an epidemiological transition characterized by extended life expectancy coupled with increasing burden of chronic, non-communicable diseases and decreasing burden of communicable diseases [6]. The disease pattern is changing as a result of marked changes in diet, reduced physical activity and other obesogenic behaviours and environmental factors. The current health care budget is already compromised with over 60% being used for the management of non-communicable diseases.

Sound evidence-based strategies are crucial in order to counteract the rising levels of obesity. There is a high rate of tracking of obesity from adolescence to adulthood [7,8] and comprehensive, multi-faceted community-based programs that start with a focus on young populations [9] are an important part of population obesity prevention. The Obesity Prevention in Communities (OPIC) project, conducted in Tonga (and Australia, New Zealand and Fiji) between 2004 and 2008, used a multilevel capacity-building approach to promote healthy lifestyles amongst adolescents [9].

The comprehensive community-based intervention program in Tonga, known locally as the Ma'alahi Youth Project (MYP), aimed to address unhealthy eating patterns and low levels of physical activity, the two major modifiable risk factors for obesity. The primary goal was to empower youth and their families to live and value healthy lifestyles. A 10-point action plan was developed and used as a road map to guide programme development and activities. The action plan consisted of three standard objectives on capacity building, social marketing and evaluation; four nutrition (promote healthy breakfast, school lunches, fruit and vegetables and water consumption); two physical activity objectives (increase organized sports and informal physical activities), and one about championing key people as role models[10]. The plan provided a framework for effective and enhanced engagement between communities, schools, and government and non-government organisations.

Process evaluation is essential to the assessment of a project's implementation, and assumes greater importance in the case of large, complex community-based intervention projects which deliver multiple, non-standardised interventions tailored to specific communities [11-13]. This paper describes the process evaluation of MYP in its mission to combat adolescent obesity. It describes the conduct of the MYP program and evaluates it in terms of the achievement of its strategic objectives, its strengths and weaknesses, and a set of best practice principles for community-based obesity prevention.

## Methods

### Staffing and Governance

Ethics approval was obtained from the Ministry of Health and Deakin University prior to project commencement. MYP was approved by the Tongan Minister of Health and endorsed by Cabinet in 2005. The project was conducted by the Fiji School of Medicine in collaboration with the Tongan Ministry of Health. It was funded by the Wellcome Trust (United Kingdom) and supported by Deakin University (Australia) and University of Auckland in terms of training, expertise and evaluation. The local project team comprised nine staff: a co-investigator, a project coordinator, three research assistants (who were responsible for the conduct of multiple community-based intervention activities), an administrative assistant, a social marketing officer and two temporary research assistants (who assisted with data collection and intervention-related activities). In addition, two managerial positions were shared by OPIC Fiji and Tonga: a study manager who provided project management in both countries, and a data manager. The Tonga Non Communicable Diseases committee advised MYP, and a management committee (comprising 12 representatives from the Ministries of Health, Education and Agriculture, schools, and non-government organisations) provided strategic direction and monitored progress.

### Participants

MYP targeted secondary school students aged 11-19 years and their families. Three intervention districts (Kolonga, Houma, and Nukunuku) on the main island of Tongatapu were selected for the intervention, comprising a total population of 2868 adolescents (according to the 1996 Census). The intervention districts comprised 22 villages and seven intervention schools (two government [public] and five church schools). Of these seven schools, three were located within and four outside the intervention districts. Those located outside were chosen because of their high enrolment of students from the intervention districts. The selection criteria for the intervention sites are further described in more detail elsewhere [9]. The comparison group consisted of six schools on the island of Vava'u, a more northerly island within the Tonga group.

### Design, development and implementation of the interventions

The project intervention and evaluation was based on a number of frameworks. A two day Analysis Grid for Elements Linked to Obesity (ANGELO) workshop [14] was conducted in February 2005 with 14 students from the intervention schools, 19 adults including representatives of the Ministry of Health, church ministers, parents, and town officers, plus 24 adolescents including youth leaders and Tonga National Youth Congress representatives. A draft 10-point Action Plan was formulated [10,15] and later modified and further developed to guide intervention activities in schools, churches and the community.

A series of data collections (a baseline survey around dietary intake and physical activity behaviour, school food audit questionnaire, community readiness assessment, sociocultural questionnaire and interviews) were conducted to inform intervention development[16]. Students were measured at baseline and follow-up in year three, with the key project outcomes being anthropometry (body mass index z-score), body fatness measured by bioelectrical impedance) and quality of life [measured by the Assessment of Quality of Life [AQoL-6D] and the Paediatric Quality of Life Inventory [PedsQL].

A research assistant was assigned to each intervention district to manage the development and implementation of the intervention program in villages, schools and churches over the period from 2006 to 2008. In each district, a committee was established to assist and support the implementation of the intervention program. In every village, a contact person was either selected by the coordinator or nominated by the community intervention committee. Schools also nominated focal points. One school appointed student ambassadors from senior forms (years six and seven, youth aged 16-18 years) to be role models for the MYP objectives.

### Process evaluation

Throughout the project, intervention-related activities were recorded by MYP staff, using a data collection proforma which included the process (i.e. how the activity was conducted), dose (i.e. scale/duration of the activity), frequency (i.e. how often the activity/event was conducted), reach (i.e. how many/type of people involved) and associated resource use (i.e. for the subsequent economic evaluation). Data were supplemented by information drawn from the MYP implementation reports, staff diaries and weekly plans, minutes of meetings, correspondence and communication with those involved in the intervention delivery on each of the strategic objectives. All intervention data were recorded on an Excel database.

Given the capacity-building focus of the MYP program, the New South Wales Framework for capacity building [17] was adopted, and its domains (partnerships, leadership, resource allocation, workforce development and organisational development) were used to structure the evaluation of this aspect of the program (Table 1). The program was also assessed (in the Discussion) against a set of Best Practice Principles developed by the Collaboration of Community-based Obesity Prevention Sites (CO-OPS) as a result of a review of the international literature and extensive community consultations[18].

**Table 1:** Capacity building objective: summary of activities

Category	Description	Objective number (#)	Comments
***Additional resources***			
Funding	13 × Grants from local and international donors	M	TOP $42,577 secured to support physical activity, vegetable gardening, training, and healthy eating activities
***Leadership***			
Students	2 × students selected for Tonga Football National Team	8	Tonga Football Association made selections after the MYP soccer competition
	2 × students attended one week WHO Ministers meeting, Auckland, New Zealand	1	Accompanied by Project Coordinator
	12 × students ambassador promoting MYP objectives		One school adopted ambassador program
	3 students attended 3 day Youth Summit		Accompanied by one research assistant. Organised by Tonga National Youth Congress
Community	16 people attended 4 day Championship Training	1	Program to train and empower community to sustain MYP intervention activities
	2 × villages developed a Community Governance Structure	1	Part of Community leadership program initiated by MYP
Staff	8 staff presentations at 6 different international conferences	1	3 staff involved
	1 staff attended 3 day Youth Stakeholders Forum	1	Training program on Youth Leadership
***Workforce Development***			
Community	12 people attended 4 community training sessions on organic vegetable gardening	1, 6	12 hours training. 2 staff also attended
	12 people attended 2 days training on drip irrigation	1	4 hours training
	76 community members attended Community Empowerment and Leadership Training × 5 days	1	4 hours × 5 days Training on Empowerment and Leadership skills × 4 villages
	12 people attended 2 hour report writing and documentation training for vegetable growing participants	1	Conducted in 3 villages
	9 young farmers attended micro-business planning workshop	1	Organized and co-hosted by MYP for young people interested in farming
Staff	2 staff attended Social Marketing Training	M, 2	1 attended 4 day workshop organised locally, the other attended a regional workshop, 2 attended 2 hour training provided locally
	2 staff enrolled in Graduate Certificate courses at Fiji School of Medicine	1,7	Epidemiology and Public Health courses
	3 staff attended 3 day Youth Task Force Workshop	1	Organized by Ministry of Training Youth and Sports
	2 staff attended 1 day Health Promoting School retreat and 3 attended 3 day Health Promoting School workshop	1	Conducted by Health Promotion Unit
	2 staff attended 32 hour basic aerobic instructor training	M	Conducted by Ministry of Health over 8 weeks
	2 staff attended 5 day EpiInfo training	1	Course delivered by New Zealand project partners
	2 staff attended one day non-government organisation leadership retreat	1	Organised by Tonga Civil Society Forum
	1 staff attended 4 day proposal and report writing workshop	1,4	Organized by Ministry of Training, Employment, Youth and Sport
	1 staff attended one day Health Promotion Foundation Workshop on governance and business plan development	1	Project coordinator attended
	1 staff attended 2 day National Youth Strategy Workshop	M	Organised by the Ministry of Training, Employment, Youth and Sport
	1 staff enrolled in Not for Profit Management course	1	Organised by Tonga Civil Society funded by New Zealand Aid
***Partnerships and collaborations***			
Government	Ongoing development of partnerships and network with 5 Government Ministries from project outset	M	Meetings and workshop held with Ministries of Health; Finance and Planning; Education; Agriculture, Food, Forestry and Fisheries; Training, Employment, Youth and Sport
Non -government organizations	Collaboration and networking on physical activity and healthy eating	M	Support in terms of funding and assistance was provided by the following: churches, schools, Youth Group, Women's Group, Farmers, Cocker Enterprise, Westpac Bank of Tonga, anonymouscdonors, Tonga Health Association, Tonga Civil Society, Tonga Trust, Tonga National Youth Congress, Development of Sustainable Agriculture in the Pacific, Tonga Communication Corporation, NZAID, AustAID, Secretariat of the Pacific Community, Food and Agriculture Organization
Organisational development	Public launch of MYP baseline results	M	Key stakeholders, school, communities, Government and Non government organisation attended.
	2 project presentations delivered locally by staff	1	One was on baseline result to community members; the other was to raise project awareness to all local health organisations

M- multiple objectives, 1- Building capacity, 2 - Social Marketing, 3 - Evaluation, 4 - Breakfast, 5 - School Lunch, 6 - Vegetables & Fruit, 7 - Physical Activity, 8 - Organized sports, 9 - Sweet Drinks vs. Water, 10 - Champions

## Results

### Evaluation by Strategic Objective

In this section, the process evaluation results set out in Tables 1, 2, 3 and 4 are discussed under each strategic objective or group of objectives - Capacity Building, Social Marketing, Nutrition (objectives around the promotion of healthy breakfasts, school lunches, fruit and vegetables and water consumption) and Physical Activity (increased organized sports and informal physical activities).

**Table 2:** Social marketing objective: summary of activities

Category	Description	Objective number (#)	Comments
***Media reports and promotions***			
Television	9 × 30 second television advertisements	5, 6, 7, 9	Developed in-house for prime time national television. Screened regularly over a six months period. Research team members used as actors.
	7 × television interviews/news items	M	Interviews with Deakin research team members aired on Tongan national television (such as at launch of baseline survey, construction of first community vegetable garden). One television program on project awareness.
Print	4 × newspaper column articles	M	MYP social marketing officer wrote column for a national newspaper
Radio	6 × 30 minute talk-back radio programs	5, 6, 7, 8, 9	National radio program comprising a talk followed by a talk-back question and answer session. Research team staff facilitated and participated in the program.
	4 × 5 minute segments in a health program	4, 6, 7, 9	Segment within a radio program run by Health Promotion Unit
	3 × 30 second radio jingles	6,7	Developed in-house for national radio. Played repeatedly.
	Radio release	M	Commencement of project
	2 × songs	M	Produced by MYP staff and aired during talk-back radio programs
***Developed materials***			
Printed	1 sign	9	Displayed in 5 locations in food shops in intervention sites
	2 sets of stickers	6, 7	1,000 stickers distributed in schools, churches, general community
	Breakfast menu	4	20 copies produced for circulation to school canteens. Developed by tertiary nutrition student on placement.
	1 × Breakfast fact sheet	4	1000x breakfast fact sheet developed and distributed into schools
	1 × video of intervention activities	M	Screened at one day OPIC symposium in Auckland, New Zealand, and also at launch of baseline results in Tonga
	1 × Project leaflet	M	Distributed to all households (in one village only) to raise project awareness
Other	2 × roadside billboards	4, 6, 7	Breakfast billboard erected in one location in each of the 3 intervention districts; other located in a central location in the capital
	11 × project banners	6, 7, 9	One outside office, others displayed at intervention events
	Project logo	M	Developed by graphic design company
	Vehicle logo	M	Used on project vehicle
	2 Food stops (sandwich display boards)	M, 6	11 boards in total displayed in front of vegetable stalls or school canteens
	T-shirts with MYP logo	M	Distributed to participants in Fun Run and Walk for Health
	1 water promotion display	9	5 Water display for display at store
***Sourced materials***			
Printed	Fat kit	4, 5, 6	Materials (including posters and other visual aids) developed by New Zealand Heart Foundation, and used for displays in schools, communities etc.
	15 × posters	4, 5, 6	Sourced from South Pacific Commission; displayed at schools or events
***Other***			
Launch	2 × project launches	M, 7	Director of Health launched MYP project; Minister for Health launched Walk for Health
	Segment in documentary on obesity	M	Filmed by Swedish crew in Tonga; research team members participated
	Free fruit distribution	6	10 fruit promotion held during inter-college sports competition and community functions
	Marketing survey on organic vegetables	6	Survey conducted with restaurants to promote availability of organic vegetables from farming project
	Survey on water promotion at a church annual conference	9	Follow up survey with on the promotion of drinking water conducted during church conference
	2 × water promotion campaigns	9	Water campaign during one week annual church conference. The other conducted at a village level. Displayed banner at both events
	World Food Day display	6, 7, 9	Displayed social marketing resources during celebrations

M - multiple objectives, 1 - Building capacity, 2 - Social Marketing, 3 - Evaluation, 4 - Breakfast, 5 - School Lunch, 6 - Vegetables & Fruit, 7 - Physical Activity, 8 - Organized sports, 9 - Sweet Drinks vs. Water, 10 - Champions

**Table 3:** Nutrition objectives: summary of activities

Category	Description	Objective number (#)	Comments
***Policies***			
	3 × schools implement food policy	4, 5, 6, 9	School with supportive canteen manager take the initiative to implement school food policy
	Organic vegetable growing governance structure developed	6	A group approach to maintain vegetable garden in the community
	3 × Food Policy workshops conducted	4, 5, 6, 9	Over a 3 year period. representatives of various government departments attended
***Activities***			
	Fruit trees planting	6	Community intervention committee initiated program to plant local fruit trees at home starting with banana tree (3 villages), hibiscus manihot tree (190 households), pawpaw and passion tree (1 village)
	1 × community breakfast	4	Demonstrated the use of local foods for breakfast
	1 × 0.5 day school canteen workshop	4, 5, 6	School canteen manager from 7 intervention schools attended
***Program***			
	4 × school vegetable gardens developed	6	To provide all year round supply of vegetables for school cooking classes. Promote student appreciation of vegetables
	18 × community vegetable gardens developed	6	Established in different villages to promote daily consumption of vegetables
	4 × markets held to sell surplus community vegetable produce	6	Surplus produce sold outside MYP work place
	8 × inspections of vegetable gardens	6	Sites visited and gardens inspected
***Infrastructure and equipment***			
	1 × school canteen established	5	Contributed to purchase of new facilities
	12 × vegetable gardens installed with drip irrigation	6	2 school garden and 10 in the community
	2 × green houses established	6	Constructed in the community
	1 × water tank repaired	9	Located in village

M- multiple objectives, 1- Building capacity, 2 - Social Marketing, 3 - Evaluation, 4 - Breakfast, 5 - School Lunch, 6 - Vegetables & Fruit, 7 - Physical Activity, 8 - Organized sports, 9 - Sweet Drinks vs. Water, 10 - Champions

**Table 4:** Physical activity objectives: activity summary

Category	Description	Objective number (#)	Comments
***Policies***			
	Nil		
***Activities***			
	1 × weekend soccer tournament organised over a 4 week period	8	All intervention sites participated plus 2 other intervention schools
	1 × weekend volleyball tournament organised over a 5 week period	8	10 × teams participated from 1 intervention community
	2 × sports competition over 3 days	7,8	3 × villages participated in different sports events
	1 × fun run event	7	Followed a 1/2 day physical activity of cleaning up the community led by youth groups
	1 × environment day	7	1 intervention site involved in a 2 hour running event
***Programs***			
	74 × one hour aerobic sessions conducted over a 1 year period	7	Targeted whole community especially adolescent girls. Conducted at village level.
	1 school conducted weekly aerobics × 2 terms	7	Targeted both students and teachers
	6 × one hour Krump sessions, 2 × action song, 2 × hip hop dance sessions conducted at Schools and community	7	Aimed to introduce students to new form of physical activity
	21 × Walk for Health events	7	Targeted whole community in different villages
	7 × one hour tennis coaching sessions	7	Targeted adolescents in one community
***Infrastructure and equipment***			
	12 × soccer balls & 8 × volley balls distributed	7, 8	Distributed to intervention schools to support organized sports. Funded by various donors.

M - multiple objectives, 1- Building capacity, 2 - Social Marketing, 3 - Evaluation, 4 - Breakfast, 5 - School Lunch, 6 - Vegetables & Fruit, 7 - Physical Activity, 8 - Organized sports, 9 - Sweet Drinks vs. Water, 10 - Programme

#### 1. Capacity Building

The Capacity Building objective aimed ***"to build the capacity of and to empower youth, their families and communities to reduce obesity through the promotion of healthy eating and physical activity among adolescents"*. **Initiatives undertaken under this objective are documented in Table 1, and are discussed below under the domains of the New South Wales Capacity Building Framework [17].

##### (a) Resources

Given limited core funding for intervention implementation, supplementary funding was sought from external donors. Despite their inexperience in preparing funding proposals, MYP staff were successful in attracting additional resources from a range of both local and regional donors to facilitate intervention activities. For example, the successful vegetable gardening project was made possible by funding obtained from the Food and Agricultural Organization of the United Nations, Secretariat for Pacific Communities and AusAID.

##### (b) Leadership

Equipping project staff, students and community leaders with leadership skills was a key factor in delivering effective interventions[19]. The consultation conducted during the project with community groups and non-government organisations revealed that lack of motivation, limited leadership skills, poor governance structures, and inadequate knowledge minimized the prospect of both the community's acknowledgement and ownership of various health related issues and of intervention sustainability. As a result, training (using a range of validated training tools such as the Pacific Star Lifeskills Training manual [UNICEF] and the Youth Leadership Training Manual [Secretariat of the Pacific Community]) was conducted in four villages to empower and strengthen the leadership skills of both individual and groups around championing healthy lifestyle strategies. Despite planning at the community level and training around healthy eating and physical activity, obesity did not rate highly on the agenda of communities. Villagers did not consider it a priority or a concern relative to more pressing issues of unemployment, poor public roads, and inadequate street lighting or water supply.

There were many concrete examples of students being empowered, as illustrated by their active participation in workshops, and their subsequent demonstration of newly acquired skills in initiating and conducting activities, preparing funding applications, and approaching stakeholders (Table 1). In one school, a school ambassador program used students as role models and motivators for change to promote better health in the school, the home and communities. Choosing students in their final year of school capitalised on their seniority but reduced the continuity of the program from one year to the next. The ambassador program was modelled on that of another OPIC site (Australia) and was, in hindsight, probably a less relevant strategy for Tonga because of the limited ability of Tongan adolescents to influence home and school environments. The other schools chose not to pursue an ambassador program but to focus on physical activity programs such as aerobics, sports competition. In a hierarchical society such as Tonga, the targeting the teachers as role models could be more successful than using students. Tonga needed to create its own strategies to fit its own peculiar situation, culture and available resources rather than adopting strategies which were effective in very different jurisdictions.

##### c) Workforce Development

Workforce development was a key component of the capacity building objective, with a range of activities conducted or attended by either staff or community members (Table 1). A particularly successful component of workforce development was the agricultural training (across both the horticultural and business aspects of farming) provided to community members in conjunction with the Development of Sustainable Agriculture in the Pacific, Ministry of Agriculture, Fisheries, Farming and Forestry, the Women's Development training centre and Ma'ui'ui Organic. The training was well received as the intervention districts rely heavily on plantation and subsistence agriculture. A large number of individuals and groups throughout Tonga (beyond the intervention districts) became interested in this agricultural program and are now involved in growing vegetables to supplement their family food supply or income generation, with some groups having progressed from small scale farming to commercial farming. There have been multiple positive outcomes, including increases in the availability and consumption of vegetables, employment and household income. The vegetable growing project has clear governance structures in place, is registered as a co-operative organization, and has nurtured an online chat room where the wider community can share the experience of developing sustainable vegetable gardens.

Given the low starting base in Tonga in terms of research and health promotion experience, the provision of opportunities for staff capacity-building was a key component. Table 1 illustrates that every opportunity was taken, within the constraints of available budget, for team staff to attend training workshops and to attend and present at forums both locally and internationally. Substantial training was also provided by OPIC staff from Australia and New Zealand primarily around topics related to the evaluation component of the projects (e.g. conduct of interviews, anthropometric measures, qualitative and quantitative analysis); evaluation is not the focus of this paper and these activities are not detailed in Table 1.

##### (d) Partnerships

Mobilizing resources and building alliances with community groups, businesses, non-government organisations, the Ministries of Agriculture, Health and Education and the mass media were instrumental to the successful implementation of the project and numerous requests for implementation of MYP activities in non-intervention communities plus invitations to participate in meetings and workshops regarding decision-making around obesity prevention (these are too numerous to detail in Table 1). For example, MYP was a key player in the drafting of the Planning Framework for the newly established Tonga Health Promotion Foundation. MYP developed particularly strong, positive and ongoing relationships with the Health Promotion Unit (within The Ministry of Health) and the Ministry of Agriculture through the Development of Sustainable Agriculture in the Pacific program, which was a key contributing factor to the success of the vegetable gardening project. However, while all groups and organizations approached by MYP were supportive, sometimes the contact was minimal, short-lived and not sustained.

#### 2. Social Marketing

The aim of MYP's social marketing component was ***"To develop a culturally sensitive, long-term national social marketing campaign to raise professional, family, and public awareness of the obesity epidemic and its effects on health and quality of life"*. **As shown in Table 2, the social marketing relied heavily on printed materials, television advertisements and radio sessions. Television and radio advertising is very cheap in Tonga, which explains the extensive use of these transmission modes to convey key messages to the target group, and to achieve wide community exposure. Another unique opportunity was to involve students as actors in the television advertisements. Talk-back radio programs attracted considerable feedback, plus a significant adult audience which was important given their role in determining the food consumed at home. Follow-up questionnaires responses suggested that the use of talk-back radio was effective.

Project branding included the development of a simple, positive project slogan "Kai Lelei mo Longomo'ui (Eat well and be active), It's so Easy!" that reflected both the nutrition and physical activity objectives of MYP. The slogan was applied to the logo, which was used on various materials (e.g. stickers, banners and television advertisements). The use of the popular rock song "It's so Easy" sung by Linda Ronstadt, gave the slogan momentum which was quickly picked up by the target audience. There was minimal focus on the print media except for a few newspaper columns and media releases around MYP activities printed in a national newspaper (which has an estimated readership of one third of Tonga's population or about 34,000 persons). Most materials were developed in-house and focused mainly on the nutrition objectives with little on physical activity.

The social marketing campaign was intended to move beyond awareness-raising to achieving behavioural change. However, it is difficult to change behaviour in a short time and in Tonga this was compounded by the fact that obesity is not considered a priority issue or a health problem. Despite this, there was some evidence of success in changing behaviours. For example, the consumption of (bottled) water significantly increased at church functions at the expense of sweetened drinks. A survey conducted at one church's annual conference over consecutive years showed that the proportion of persons drinking water during the conference rose from 75% to 85%; this was attributed to MYP mounting a water campaign during that event which included television advertisements (that used church leaders and students as key players), talk-back radio programs, a banner displayed during church conferences and water promotion displays in shops located in the intervention sites.

#### 3 Nutrition

The nutrition objectives focused on the promotion of a healthy breakfast (objective 4), healthy school lunches (objective 5), vegetable and fruit consumption (objective 6), and water versus sweetened drinks (objective 9). While most intervention activity was focussed on vegetable gardening given strong partnerships with other agencies and specific funding by various donors (Table 3), all four nutrition objectives were addressed by the social marketing campaign (Table 2). The nutrition objectives are discussed under headings which correspond to Table 3.

##### (a) Policy

The nutrition component of the project primarily focused on grass-roots activities, with little done in the way of developing nutrition policy. The team lacked the skills and experience to drive policy change using top-down approaches but later realised that effective obesity prevention strategies needed to be embedded in well-established policy. School food policy designed by the Ministry of Health and modified by the Non-Communicable Diseases Nutrition Sub-committee was introduced into three intervention schools where the canteen manager was supportive. These policies needed further refinement at the school level as most healthy food is expensive and not affordable by students. Also, a dedicated person was required within a school to drive policy implementation.

##### (b) Activities

Activities refer to one-off initiatives or events while programs relate to ongoing initiatives. Amongst the activities initiated, fruit tree planting demonstrations were held in 16 of the 22 villages in the intervention districts, while, given funding constraints, healthy breakfast demonstrations (based on local, affordable, traditional food) were limited to one village which specifically requested such. Activities around food at school were limited to the establishment of a new canteen in one school and the conduct of a canteen operators' workshop for the seven intervention schools. The latter did not result in a change in the healthiness of the food sold, thereby illustrating the importance of a top-down approach, the development of partnerships with schools, and the need for a designated person within the school to steer change.

With respect to the objective of promoting water rather than sugar sweetened drinks, most of the activities took place in association with the physical activity and social marketing programs. Considerable effort was invested in the promotion of water, during the one week Free Wesleyan Church of Tonga annual conference. The church leaders were encouraged to act as role models by providing only water for drinking during all conference meals. This was endorsed by the First Secretary of the Church and letters were distributed to all community church leaders to inform them of this decision. People's attitudes to this initiative were positive and promising. The church maintained the water only policy at their next annual conference and several other churches and villages followed with similar policies. The significance of this achievement cannot be downplayed in a culture where food is a status symbol. It is a sign of wealth and caring and a source of pride for Tongans to provide their guest with copious quantities of food and drink which is often very high in sugar and saturated fats. The success of the church program reflects the importance of a top-down approach in churches as well as in schools.

##### (c) Programs

The growing of vegetables was a key plank of the nutrition objectives. The main strategies were to make vegetables available for a community's own consumption and to simultaneously improve eating habits. The gardens were located in close proximity to the villages to facilitate resident access. Despite a lack of previous agriculture experience, participants were enthusiastic and committed. All group members were involved right from the outset in planning through to implementation and distribution of produce, and participated in various training opportunities offered by different organizations in relation to vegetable gardening (Table 1). Technical support for the program was provided through involvement of agricultural and marketing personnel from the Ministry of Agriculture, and both local and international donor support was secured. All participants were required to sign a Memorandum of Understanding around the program objectives and expectations around promoting health. In addition to the development of 20 new family gardens within the intervention communities, there has been a multiplier effect beyond the intervention site with the establishment of new gardens. Interested groups from schools, churches, villages and youth groups have now formed committees (including representative of the Ministries of Health and Agriculture) to ensure the ongoing conduct of the program.

##### (d) Infrastructure and equipment

As part of the vegetable gardening activities, two green houses were erected, and drip irrigation was installed in 13 vegetable gardens.

#### 4. Physical Activity

Physical activity objectives in the action plan were to increase participation in informal physical activities (objective 7) and organised sports (objective 8). Activities organised under this programme ranged from aerobics, walks for health, volleyball, tennis, cleaning up of communities, soccer, hip hop, cultural dancing, krump, action song, fun runs and sports competitions. The amount of activity generated under the category of physical activity was considerable (Table 4).

However, while these programs were enjoyed very much by the community, they were not sustainable hence their impact was short-lived. The intervention team was unable to keep up with the heavy demand for physical activity programs, and the lack of funding did not allow proper training of instructors from villages and schools. An aerobics instructors' training course was conducted, but failed to produce ongoing instructors given the lack of continuing support available to them. In hindsight, more forward planning around the sustainability of physical activity activities and the choice of activities would have aided integration of the programs into existing structures. The physical activities program was conducted primarily at the village level and attracted the whole of the community; while this was beneficial in itself, the impact on the adolescent target group was likely to be diluted. Physical activity programs directed towards the secondary schools instead of communities may have been more sustainable.

## Discussion

The MYP project implemented a large number of activities and initiatives over the three year period. In this section, the program is discussed using the best practice principles developed for community-based obesity prevention initiatives [18].

### (a) Community engagement

Before any community-based intervention is implemented, a planned approach to community engagement is important [19]. It requires genuine community participation from the beginning: identification and acceptance of the problem, agreement that it needs to be addressed, an understanding of the target group's situation, and the engagement of influential people in the community as well as parents, youth, community groups, churches etc. Whole-of-community (churches, schools, parents, leaders, adolescents) engagement and collaborative partnerships were critical to the implementation of the MYP project. In the early stages, the coordinators visited their designated intervention site multiple times to familiarize themselves with the community setting. Meetings were organised with key people such as village leaders and the community health officer, whose support was vital if the community was to be engaged. In hindsight, more time was needed for this phase of community engagement so that the interventions better matched communities.

Monetary incentives are commonly used by projects to elicit community engagement in Tonga; many externally run projects pay people to participate in surveys and programs. Payment for involvement was often raised but MYP staff endeavoured to convince adults that the project priority was their children's health and they would benefit from this program. The policy of no monetary payments did make the team's task of engendering community support and commitment more challenging. The level of community engagement secured varied throughout the intervention sites. In some villages, the complexity of communal living made it difficult for the project to introduce strategies, while other villages welcomed any initiatives offered. A commonly heard phrase in discussion with the communities when trying to win them over to lead a healthier lifestyle was "eating is mine to decide upon, diseases is for the doctors to take care of". This reflective of community attitudes towards obesity; the fact that it was not considered a problem made the project team's task more difficult.

### (b) Program design and planning

Program design and planning was under pressure because of delays in hiring MYP staff and pressure to match the schedule of the other three OPIC sites. The development phase of the social marketing campaign provides an example where pressure to proceed with interventions meant that the baseline survey results were not available in time to inform the strategy. As a result, MYP sometimes introduced initiatives that matched available staff resources and expertise, rather than building on community needs or preferences. Some implemented programs would have benefited from more planning to ensure delivery suited specific community situations, the adolescent target group, village's socio-economic status, geographical location and cultural values. For instance, early initiatives to promote active transport to school were unrealistic as most students in the target group travelled long distances by bus to school in Nuku'alofa. Likewise, while the preparation of local foods for breakfast was encouraged, this was impractical as most students left home early to catch the one and only bus service to school. On the other hand, the vegetable farming project was highly relevant in the Tongan context given that farming is part of the everyday life of most Tongans.

### (c) Implementation and sustainability

Intervention activities were implemented at various levels, with multiple audiences and in multiple locations; the limited resources (staff and funds) coupled with the differing levels of community engagement inevitably meant that the intervention dose was not necessarily equal across all 22 villages and seven schools. Also when MYP project activities were initiated in the intervention districts, government agencies sometimes withdrew any current activities around healthy eating and physical activity and redirected their attention to non-intervention sites.

Although the target group of the MYP project was secondary school students, most activities were conducted at the village community level with all age groups. However, program impacts beyond adolescents were not captured and the intervention dose to adolescents was diluted by such whole-of-community programs. Overall, the intervention dose was probably inadequate as health promotion programs typically take a long time to mature and for the benefits to be realised. The 2.5 years of MYP meant that the intervention was still maturing when the project funding ceased. For example, the social marketing officer who offered the capacity to galvanise social mobilization was recruited late in the project; and the implementation of the interventions was severely interrupted both by a long period of mourning following the death of the Tongan king and a period of civil unrest.

Tonga is a church-oriented society with a high proportion of the population regularly participating in church activities. In retrospect, faith-based organisations could have been more involved in delivering MYP messages and capacity building activities.

### (d) Governance and accountability

The successful conduct of a large, multi-faceted community-based intervention project such as MYP is reliant, in part, on having a project coordinator who is confident, committed, motivated, has a strong ability to network, and has the leadership skills to manage a multi-disciplinary team and to coordinate multi-sectoral action with other stakeholders, multiple communities and settings. Governance needs leadership, alliances, and staff expertise. All were a challenge because of Tonga's low professional capacity and its inexperience in health promotion and research. A strong alliance with professionals from other sectors such as education, agriculture, labour and commerce and non-government organizations is also required. Staff recruitment around specific areas of the project (eg. nutrition, physical activity) together with supporting roles from others may have been a more effective model than research assistants being assigned to geographical areas and being expected to embrace all facets of the project.

The process evaluation reported in this paper is necessarily different from that of other projects reported in the literature [11-13]. The latter process evaluation papers generate relate to the delivery of a standardised intervention. However, in the case of MYP, intervention activities were much less structured, and were organic and evolving. Activities varied between schools and villages depending on their own specific needs and interests; as a result, the range of different activities undertaken was large. Our process evaluation was, as a consequence, limited to dose, frequency and reach, and did not cover other issues such as fidelity and quality used in other process evaluations.

The key lessons learnt from the process evaluation of MYP are as follows:

• Intervention activities should suit the local cultural context and be tailored to community needs.

• Top-down policy approaches are crucial in all settings.

• Adequate time needs to be allocated for planning and community engagement prior to embarking on intervention activities.

• Social marketing is fundamental to project success but is potentially time-consuming and expensive.

• Successful community-based projects are dependent on sustained alliances with a wide range of players.

## Conclusions

The adolescent obesity interventions implemented as part of the MYP program comprised a wide range of activities conducted in multiple settings, touched a broad spectrum of the population (wider than the target group), but the dose and frequency of activities were generally insufficient and not sustained. While the project resulted in increased community awareness of healthy behaviours, it confirmed that Tonga is still in its infancy in terms of conducting public health research and lacks research infrastructure and capacity.

Despite its limitations, the MYP project was an extremely beneficial and relevant study for Tonga given its high level of obesity and non-communicable diseases. It should be considered as a base on which to further build and strengthen future public health research in Tonga, particularly around obesity prevention. Since this is the first large, multi-faceted community-based project of its kind in Tonga, it is important to take learning from both of its achievements and failures. Effective programs require good leadership, comprehensive planning, widespread community engagement, multiple approaches, multiple partnerships, extensive capacity building, adequate intervention dose and most importantly, community-based initiatives designed, implemented and monitored in a culturally context perspective.

## Authors' contributions

KFF was involved with data collection, implementation of project activities, analysis and interpretation of results, and drafting of the first manuscript. MLM contributed to the design and evaluation of the program and its activities and was closely involved in drafting the manuscript and critically reviewing it in terms of intellectual content. HMM reviewed the manuscript for errors and important intellectual content. SP conducted the social marketing component of MYP and contributed to this section of the paper. JTS was involved with the design and development of MYP and editing of the manuscript. BAS participated in the designing of the study and critical review of the manuscript.

## Pre-publication history

The pre-publication history for this paper can be accessed here:

http://www.biomedcentral.com/1471-2458/11/284/prepub
